# Predicting externalizing symptom trajectories in U.S. National Guard recruits: The role of adverse childhood experiences

**DOI:** 10.1002/jts.70037

**Published:** 2025-12-24

**Authors:** Ali F. Sloan, Tristan Bron, Craig A. Marquardt, Seth G. Disner, Siamak Noorbaloochi, Melissa A. Polusny, Jonathan D. Schaefer

**Affiliations:** ^1^ Department of Psychology Vanderbilt University Nashville Tennessee USA; ^2^ Department of Psychology Liberty University Lynchburg Virginia USA; ^3^ Minneapolis VA Health Care System, 1 Veterans Drive Minneapolis Minnesota USA; ^4^ Department of Psychiatry and Behavioral Science University of Minnesota Minneapolis Minnesota USA; ^5^ Center for Care Delivery Outcomes Research Minneapolis Minnesota USA

## Abstract

Adverse childhood experiences (ACEs) are strongly associated with increased risk of externalizing problems. Despite their prevalence in military populations, limited research links ACEs to longitudinal externalizing problem trajectories during military service transition. This study aimed to identify distinct trajectories of externalizing problems (deviant behavior, alcohol use, and drug use) in U.S. Army National Guard recruits and examine how baseline ACEs predict membership in higher‐risk trajectories during the transition to military service. A longitudinal cohort of 707 Army National Guard recruits was assessed before basic combat training and at four follow‐ups over 18 months. Growth mixture modeling was used to identify distinct trajectories for deviant behavior, alcohol use, and drug use, whereas logistic regression analyses were conducted to examine associations between baseline ACEs and trajectory group membership. For each domain, we identified distinct trajectory patterns beyond stable‐low: decreasing‐increasing and increasing‐decreasing deviant behavior trajectories, stable‐high and increasing alcohol use trajectories, and a variable drug‐users trajectory. Relative to stable‐low class membership, higher ACE scores were associated with increased odds of membership in the decreasing‐increasing, *OR* = 1.26, 95% CI [1.12, 1.41], and increasing‐decreasing, *OR* = 1.26, 95% CI [1.15, 1.37], deviant behavior; stable‐high alcohol, *OR* = 1.13, 95% CI [1.03, 1.25]; and drug‐users, *OR* = 1.19, 95% CI [1.11, 1.28], trajectories. Specific ACEs uniquely predicted higher‐risk trajectories. These findings suggest that ACEs may have longitudinal effects on the unfolding of externalizing symptom trajectories among military recruits, highlighting the need to address preexisting developmental vulnerabilities when examining pathways to psychopathology during significant life transitions.

Childhood adversity, defined as negative disruptions to rearing environments that require significant adaptation from children to maintain functioning (McLaughlin, 2016), can exert profound and detrimental effects across various developmental domains (Cicchetti & Toth, 2016). Traditionally, adverse childhood experiences (ACEs) include physical, sexual, and emotional abuse; physical and emotional neglect; and household dysfunction (i.e., domestic violence, parental separation or divorce, substance use, psychopathology, and incarceration), all of which are highly interrelated (Dong et al., 2004). ACEs are potent risk factors for psychopathology (McLaughlin, 2016) and are associated with stress response dysregulation that may increase vulnerability to externalizing behaviors (al'Absi et al., 2021).

A growing literature supports an association between ACEs and an increased risk for externalizing behaviors (Leban, 2021; Muniz et al., 2019). The cumulative risk model—the most common framework for conceptualizing adversity—sums the total number of distinct ACEs, creating an individual risk score to predict outcomes (Evans et al., 2013; Felitti et al., 1998). Evidence suggests that ACE exposure increases externalizing risk in a dose–response relationship, with higher scores linked to higher risk (Schilling et al., 2007).

However, recent evidence suggests specific adversities are differentially associated with distinct outcomes. For example, household dysfunction may be more strongly associated with externalizing symptoms than abuse (Benjet et al., 2010; Gomis‐Pomares & Villanueva, 2023; Muniz et al., 2019), potentially reflecting a manifestation of the genetic liability for externalizing pathology. However, physical and emotional abuse have also emerged as significant predictors of externalizing symptoms (Gomis‐Pomares & Villanueva, 2023; Muniz et al., 2019). When examining specific problems, abuse forms are consistently linked with alcohol‐related problems (Kisely et al., 2020), whereas both abuse and neglect are associated with delinquent behaviors (Degli Esposti et al., 2020; Nkuba et al., 2019).

Military populations present a unique context for examining ACE effects, as military service demands may shape developmental trajectories for individuals with a history of adversity. Veterans report higher rates of nearly all ACE forms compared to nonveterans (Blosnich et al., 2021). Further, most U.S. military personnel enlist during emerging adulthood (Mobbs & Bonanno, 2018), a period characterized by identity exploration and increased risky behaviors (Arnett, 2016; Moffitt et al., 2002; Schulenberg et al., 2018). However, individuals with a history of ACE exposure often adopt adult roles earlier, potentially bypassing this explorative stage (Arnett, 2016). Military service may offer structured pathways to adult status and independence, with life course theories suggesting such turning points can redirect these developmental trajectories (Sampson & Laub, 1993).

ACEs are strongly associated with a range of mental health challenges among service members, including posttraumatic stress disorder (PTSD), anxiety, depression, and suicidal ideation (Blosnich et al., 2021; Davis et al., 2022; Slavin et al., 2024), even after adjusting for combat exposure (Aronson et al., 2025). Moreover, civilian trauma has been associated with increased PTSD and depression risk in reservists, suggesting nonmilitary adversity plays a central role in shaping psychopathology among U.S. National Guard personnel (Fink et al., 2016; Polusny et al., 2011). However, military mental health research has largely centered on deployment‐related internalizing psychopathology and postservice transition (Mobbs & Bonanno, 2018), leaving gaps in understanding externalizing behaviors during early service, when adjustment challenges often arise. Evidence from the Australian Defence Force shows psychological distress symptoms frequently emerge early in military careers, even without deployment experiences (Dell et al., 2023), underscoring the importance of studying this formative period.

Research linking ACEs to externalizing problems in military populations remains limited. Increased exposure to childhood adversity has been associated with greater substance‐related problems in military samples (Davis et al., 2022; Murphy & Turgoose, 2022; Zheng et al., 2016). More specifically, emerging evidence suggests that childhood sexual abuse and household substance use predict alcohol problems, whereas sexual abuse and household incarceration predict drug problems (Clarke‐Walper et al., 2014; Moore et al., 2024). These findings suggest that specific ACEs may differentially increase the risk of distinct externalizing outcomes, highlighting the need for further investigation in military populations, particularly regarding non–substance‐related externalizing behaviors. Further, taking a dual approach that examines both the total burden of adversity (cumulative risk) and the impact of specific experiences is critical. This approach can clarify whether universal, broad‐based support is sufficient or if targeted interventions aimed at mitigating the consequences of specific types of childhood adversity are necessary to prevent maladaptive outcomes during the transition to military service.

The U.S. Army National Guard offers a distinct and understudied context for examining the link between ACEs and externalizing behaviors. Although they complete the same initial training as their active component counterparts, National Guard soldiers balance part‐time military service with civilian employment. These dual roles, compounded by unpredictable deployments, may induce more psychological strain than stable active duty environments (Cohen et al., 2015). Spending a majority of time in civilian settings after the initial training period might increase vulnerability to risky behaviors compared to supervised active duty counterparts. Despite identical military regulations, reservists in the United States and elsewhere show higher drug‐related offense rates (Gloyd & Leal, 2019) and problematic alcohol consumption (Diehle et al., 2021) compared to active duty personnel, suggesting military service may provide less consistent protection against externalizing problems—particularly substance‐related problems— for reservists (Salvatore & Taniguchi, 2021).

Understanding these dynamics is critical, as externalizing behaviors can result in disciplinary actions and discharge from service. Attrition across military branches ranges from 18.5% to 29.7% by 36 months (Marrone, 2020), representing a significant loss of personnel and training investment. Mental health concerns are among the leading causes of early separation across military branches (Larsson et al., 2009). Supporting the role of preservice vulnerabilities, U.S. Marine Corps recruits with premilitary interpersonal trauma were found to be 1.5 times more likely to drop out of training than those who had not experienced such trauma (Wolfe et al., 2005). However, no research has investigated how early adversity shapes the developmental course of externalizing behaviors in National Guard members during the early stages of service, an important gap given the implications for retention.

The present study addressed key literature gaps by examining how ACEs predict externalizing symptom trajectories among U.S. National Guard recruits during early military service. By focusing on emerging adults during this critical developmental transition, we captured a period of significant adjustment that may be particularly influenced by early adversity. We hypothesized that higher cumulative ACE scores would be associated with membership in elevated or increasing externalizing symptom trajectory classes, with associations specific to abuse‐related ACEs. The longitudinal design enabled us to capture behavioral changes over the first 18 months of service, offering insight into how behaviors evolve across a pivotal developmental transition. By integrating cumulative and individual risk models within a person‐centered framework, this study offers implications for emerging adults navigating transitions and military policies enhancing adaptive functioning during service.

## METHOD

### Participants and procedure

We analyzed data from the Advancing Research on Mechanisms of Resilience (ARMOR) longitudinal cohort study. Study protocol and data collection procedures have been described previously (Polusny et al., 2023). Briefly, a sample of Minnesota Army National Guard recruits (*N* = 1,201) completed a large battery of assessments before basic combat training (BCT) and at four follow‐up assessments: within 2 weeks of completing training, 6 months later, 12 months later, and 18 months later. Study measures included computerized self‐administered questionnaires at each time point. All participants provided informed consent. Data were deidentified, and participants were assured that their individual responses would be kept confidential. Institutional Review Board approval was obtained from both the University of Minnesota and the Minneapolis VA Health Care System before study onset, and the study adhered to American Psychological Association (APA) standards regarding the ethical treatment of participants, informed consent, and confidentiality.

The analytic sample was limited to participants who provided ACEs data at baseline, were exposed to BCT, were eligible for study follow‐up, and completed externalizing symptom measures at baseline and at least one follow‐up assessment. After exclusions, the final sample consisted of 707 participants, with a mean age at baseline of 18.9 years (*SD* = 2.8, 71.4% male; 62.7% White). A detailed overview of the study flow, including participant exclusions and retention, is provided in the CONSORT diagram (Supplementary Figure S1). Of the 707 participants with baseline data, sample sizes at each time point were 667 at post‐BCT, 583 at 6‐month follow‐up, 548 at 12‐month follow‐up, and 536 at 18‐month follow‐up.

### Measures

#### ACEs

Exposure to childhood adversity was assessed at baseline using a combination of the Adverse Childhood Experiences Questionnaire (Felitti et al., 1998) and the Deployment Risk and Resilience Inventory‐2, Prior Stressors Scale (DRRI‐2; Vogt et al., 2013). The ACE Questionnaire is a widely used 10‐item measure assessing exposure to emotional, physical, and sexual abuse; emotional and physical neglect; and household dysfunction (including parental substance misuse, mental illness or suicidality, incarceration, and domestic violence exposure) during the first 18 years of life. Following child protection recommendations, the ACE Questionnaire item assessing domestic violence exposure was omitted from the current study. Instead, we utilized an item from the DRRI‐2 Prior Stressors Scale, which assesses lifetime exposure to stressful events and violence, including domestic violence (“I saw or heard physical fighting between my parents or caregivers”). Given that the sample's average baseline age was approximately 18 years, this item provided an appropriate assessment of domestic violence exposure during childhood. This substitution maintained assessment of all 10 ACE domains in the final analyses, consistent with the original ACE framework. Each domain was coded as a binary (0 = no exposure, 1 = exposure), and a cumulative ACE score was calculated (range: 0–10).

#### Externalizing behaviors

##### Deviant behavior

Deviant behavior was assessed using items adapted from the Behavioral Report on Rule‐Breaking (BHR; Nelson et al., 2011) and two items from the Revised Conflict Tactics Scale (CTS; Straus & Douglas, 2004). The BHR is a 33‐item measure that evaluates an individual's propensity to engage in behaviors that violate established rules and norms (e.g., “How often did you go onto someone's property when they did not want anyone there?,” “Not counting a fight with your brother or sister, how often did you hit someone hard enough for him or her to need bandages?”) while drawing items from several other published measures (Clark & Tifft, 1966; Hindelang et al., 1981; Nye & Short, 1957). To reduce participant burden, only 18 items from the BHR were administered. The CTS assesses the frequency of conflict and violence between respondents and romantic partners (e.g., “How often did you push, shove, or slap a romantic partner [i.e., a boyfriend or girlfriend]?”). Scores were summed to create a total deviant behavior score (range: 0–60). If more than 20% of items on the measure were missing, a total score was not calculated; for cases with 20% or fewer missing items, missing values were imputed using the participant's mean item score. Cronbach's alpha for the combined measure at baseline was .77, indicating acceptable internal consistency.

##### Alcohol use

Alcohol use was assessed using the Alcohol Use Disorders Identification Test (AUDIT; Saunders et al., 1993), a 10‐item screening instrument for distinguishing hazardous from nonhazardous alcohol use (e.g., “How often do you have a drink containing alcohol?,” “How often during the last year have you found that you were not able to stop drinking once you had started?”). Each item is scored on a 0–4 scale, with total scores ranging from 0 to 40 and scores of 8 or higher indicating hazardous alcohol consumption. If more than 20% of items on the measure were missing, a total score was not calculated; for cases with 20% or fewer missing items, missing values were imputed using the participant's mean item score. Cronbach's alpha at baseline was .81, reflecting good internal consistency.

##### Drug use

Drug use was assessed using the 10‐item Drug Abuse Screening Test (DAST‐10; Skinner, 1982), with total scores ranging from 0–10 and scores of 3 or higher indicating a moderate risk of drug‐related problems. The DAST‐10 includes a “skip‐out” pattern such that respondents who answer “no” to the first item (“In the past 12 months, have you used drugs other than those required for medical reasons?”) do not complete the remaining items and are assigned a total score of 0. For participants who endorsed the first item, if more than 20% of the remaining items were missing, a total score was not calculated; if 20% or fewer items were missing, missing responses were imputed using the participant's mean item score. Cronbach's alpha at baseline was .53 among participants who endorsed the first item; this poor internal consistency likely reflects low endorsement in the current sample, as prior research has demonstrated good internal consistency (Cronbach's α = .86; Yudko et al., 2007).

#### PTSD

Symptoms of PTSD were assessed at baseline using the Primary Care PTSD Screen for *DSM‐5* (PC‐PTSD‐5; Prins et al., 2016), a five‐item screener tied to criteria outlined in the *Diagnostic and Statistical Manual of Mental Disorders* (5th ed.; *DSM‐5*; American Psychiatric Association, 2013). Respondents indicate whether they have experienced each symptom in the past month (“yes” or “no”), with endorsed items scored as 1, and total scores range from 0 to 5. In this sample, internal consistency was acceptable, Cronbach's α = .79.

### Data analysis

The analytic sample was limited to participants who provided ACEs data at baseline, were exposed to BCT, were eligible for study follow‐up, and completed externalizing symptom measures at baseline and at least one follow‐up assessment (*N* = 707).

Complete methodological details and theoretical rationale for our trajectory analysis approach are provided in the Supplementary Materials. Briefly, we first established the optimal unconditional growth model for each domain, assuming a single class. Using this best‐fitting unconditional model as a base, we then employed growth mixture modeling (GMM) to estimate trajectory class parameters for each externalizing behavior domain using the *lcmm* package in R (Proust‐Lima et al., 2017), testing solutions with one to four classes. Model selection was guided by fit indices, including the Bayesian information criterion (BIC) and Akaike information criterion (AIC), as well as clinical interpretability. Given uncertainty in class assignment, we used estimated posterior probabilities of class membership to generate 10 stochastically imputed class assignments for each participant rather than using a singular deterministic (most‐likely) class assignment. We then examined associations between ACEs and each of the 10 imputed class memberships using multinomial logistic regression from the *nnet* package in R (Venables & Ripley, 2002) and reported pooled results using Rubin's rules. For each externalizing behavior, we first designated the lowest‐risk trajectory class as the reference, then conducted additional analyses with alternative reference groups to enable direct comparisons between higher‐risk trajectory classes. We tested (a) cumulative ACE scores predicting class membership, (b) each individual ACE predicting class membership in separate models, and (c) simultaneous models with all ACEs entered together predicting class membership. Model results were adjusted for age, sex assigned at birth, and race. All analyses were conducted in R (Version 4.4.1).

As a sensitivity analysis, we reestimated all multinomial logistic regression models, controlling for baseline PTSD symptoms. Although PTSD is unlikely to causally precede ACEs, it may increase retrospective reporting, so we included PTSD as a covariate to provide a conservative test of whether ACE–externalizing associations persisted after accounting for potential recall bias due to symptoms subsequent to ACE exposure. However, it should be noted that because PTSD may also partially mediate these associations, this adjustment could lead to overadjustment and, thus, yield conservative effect estimates.

## RESULTS

### Descriptive characteristics

ACE exposure was prevalent in our analytic sample, with 71.9% of participants reporting exposure to at least one ACE. The average cumulative ACE score was 2.5 (*SD* = 2.5). The percentage of the sample exposed to each specific ACE is presented in Supplementary Figure S2 Descriptive statistics for externalizing behaviors at each assessment wave are presented in Table 1.

**TABLE 1:** Externalizing symptoms over time

	Deviant behavior				Alcohol use				Drug use			
Timepoint	*N*	*M*	*SD*	Range	*N*	*M*	*SD*	Range	*N*	*M*	*SD*	Range
Pre‐BCT	705	2.78	3.61	0–32	702	2.70	3.29	1–26	706	0.23	0.82	0–7
Post‐BCT	663	2.23	4.09	0–41	660	2.04	3.68	0–25	666	0.18	0.86	0–8
6‐month follow‐up	583	2.67	5.29	0–54	576	2.62	4.74	0–32	582	0.18	0.97	0–10
12‐month follow‐up	545	2.76	5.22	0–36	542	2.92	4.8	0–34	544	0.28	1.16	0–10
18‐month follow‐up	536	2.42	4.53	0–34	530	2.88	4.36	0–30	534	0.19	0.91	0–10

*Note*: All values are calculated based on the analytic sample. *N* values represent the number of participants with complete data. BCT = basic combat training.

### Model selection

**FIGURE 1:**
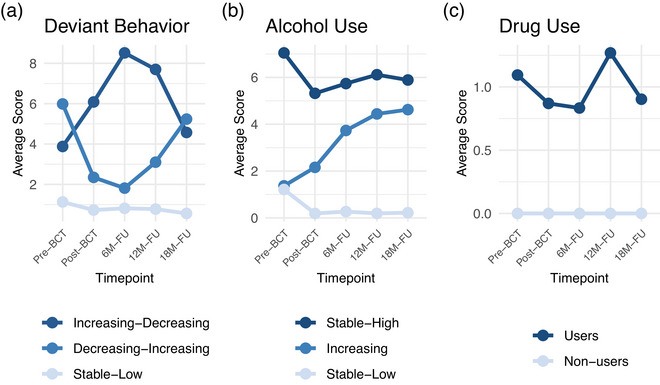
Externalizing symptom trajectories *Note*: The figure depicts mean symptom scores for each identified trajectory group across the five assessment time points—pre–basic combat training (BCT), post‐BCT, 6‐month follow‐up (6M–FU), 12‐month follow‐up (12M–FU), and 18‐month follow‐up (18M–FU)—for (A) deviant behavior, (B) alcohol use, and (C) drug use.

For each externalizing symptom domain, we first identified the best‐fitting unconditional growth model (Supplementary Table S1), which served as the baseline structure for evaluating one‐to‐four‐class GMMs. Across all domains, multiclass solutions demonstrated superior fit, supporting the use of mixture modeling (Supplementary Table S2). Complete model fit statistics and detailed selection rationale are reported in the Supplementary Materials. The estimated trajectories for the final selected models are presented in Figure 1.

#### Deviant behavior

A three‐class quadratic model provided optimal fit, BIC = 3,916, and clinical interpretability, as well as adequate classification certainty, entropy = .619. This model identified (a) a stable‐low trajectory (56.4% of participants), characterized by consistently minimal deviant behavior across all time points; (b) a decreasing‐increasing trajectory (21.4%), showing an initial decrease in deviant behavior followed by escalation over the observation period; and (c) an increasing‐decreasing trajectory (22.2%), demonstrating initial escalation of deviant behavior that then diminished over the observation period.

#### Alcohol use

A three‐class quadratic model yielded the best BIC (3,403) and good classification certainty, entropy = .758. This model identified (a) a stable‐low trajectory (45.6% of participants), characterized by consistently minimal alcohol consumption across all time points; (b) an increasing trajectory (29.8% of participants), showing gradual escalation in drinking patterns over time; and (c) a stable‐high trajectory (24.6% of participants), characterized by consistently elevated alcohol use compared to the majority of the sample across all time points.

#### Drug use

A two‐class quadratic model provided optimal fit (BIC = −1,726) and excellent classification certainty (entropy = .974). This model identified (a) a nonusers trajectory (78.9% of participants), characterized by no drug use across all time points, and (b) a drug users trajectory (21.1% of participants), characterized by some degree of drug engagement over time.

### Cumulative ACE exposure as a predictor of externalizing symptom trajectories

**FIGURE 2:**
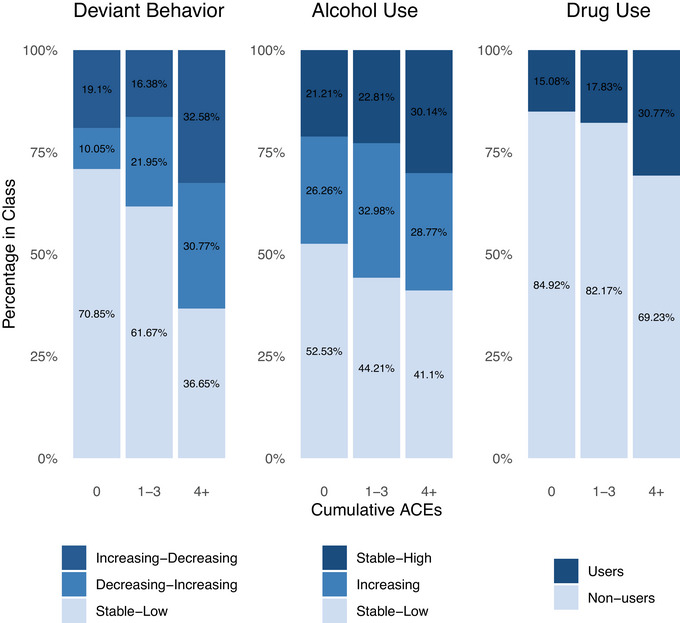
Distribution of trajectory class membership by cumulative adverse childhood experiences (ACEs) *Note*: The figure depicts the percentage of the analytic sample in each trajectory class by exposure to cumulative ACEs (i.e., the number of different ACE types endorsed at baseline).

Cumulative ACE exposure significantly predicted class membership across all three externalizing domains. For deviant behavior, multinomial logistic regression indicated that higher degrees of ACE exposure were associated with higher odds of belonging to both the decreasing‐increasing, odds ratio (*OR*) = 1.26, 95% confidence interval (CI) [1.12, 1.41], *p* <.001, and increasing‐decreasing trajectories, *OR* = 1.26, 95% CI [1.15, 1.37], *p* < .001, relative to the stable‐low class. A direct comparison between the increasing and decreasing classes showed that ACE exposure was not significantly associated with differential odds of class membership, *p* = .999. For alcohol use, multinomial logistic regression showed that higher levels of ACE exposure were linked to higher odds of belonging to the stable‐high trajectory, *OR* = 1.13, 95% CI [1.03, 1.25], *p* = .012, but not the increasing trajectory, *OR* = 1.04, 95% CI [0.95, 1.13], *p* = .424, relative to the stable‐low class. A direct comparison between these two higher‐risk classes showed that higher ACE exposure was associated with marginally significantly higher odds of stable‐high versus increasing trajectory membership, *OR* = 1.09, 95% CI [1.00, 1.20], *p* = .050. For drug use, multinomial logistic regression indicated that higher ACE exposure was associated with higher odds of belonging to the drug‐users trajectory, *OR* = 1.19, 95% CI [1.11, 1.28], *p* < .001, relative to the nonusers trajectory. The percentage of the sample in each deterministic (i.e., most likely) class by cumulative ACE exposure is visualized in Figure 2.

### Individual ACEs as predictors of externalizing symptom trajectories

**FIGURE 3:**
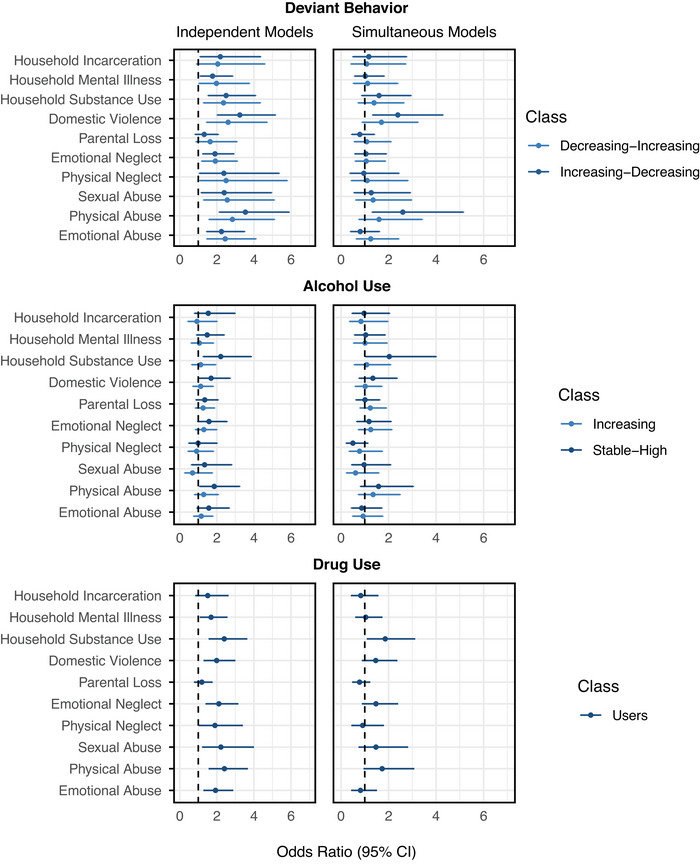
Associations between individual adverse childhood experiences (ACEs) and externalizing symptom trajectories *Note*: The figure depicts the results from models examining the association between ACEs and trajectory class membership, with the stable‐low trajectory as the reference group. The left column presents results from independent models, where each ACE was entered individually, whereas the right column presents results from simultaneous models, where all ACEs were entered together. Values greater than 1.0 indicate a higher likelihood of trajectory group membership associated with the corresponding ACE. CI = confidence interval.

The results from the separate models with ACEs each entered individually are shown in Figure 3. For deviant behavior, all ACEs except parental loss were associated with higher odds of belonging to the increasing‐decreasing trajectory, and all ACEs except parental loss and incarceration were associated with higher odds of belonging to the decreasing‐increasing trajectory, relative to the stable‐low class. No specific ACEs predicted membership in the increasing‐decreasing class compared to the decreasing‐increasing class. For alcohol use, exposure to physical abuse, domestic violence, and substance use in the household were all individually associated with higher odds of belonging to the stable‐high trajectory relative to the stable‐low class. Of note, no ACEs predicted membership in the increasing class compared to the stable‐low class, but a direct comparison of the higher‐risk trajectories showed substance use in the household was associated with increased odds of belonging to the stable‐high trajectory relative to the increasing trajectory, *OR* = 1.96, 95% CI [1.12, 3.43], *p* = .019. For drug use, all ACEs except parental loss and parental incarceration were individually associated with higher odds of belonging to the drug‐users trajectory relative to the nonusers class.

### Simultaneous models examining the distinct effects of ACEs on externalizing symptom trajectories

The results from the models in which all ACEs were entered simultaneously are also shown in Figure 3. Prior to estimating these models, we examined intercorrelations and variance inflation factors (VIFs), which indicated that multicollinearity was not a concern, *r*s = .13–.64, VIFs = 1–2. For deviant behavior, exposure to physical abuse and exposure to domestic violence were associated with higher odds of belonging to increasing‐decreasing class relative to the stable‐low class, whereas no individual ACEs significantly predicted membership in the decreasing‐increasing trajectory when controlling for all other ACEs. No ACEs predicted membership in the increasing‐decreasing class compared to the decreasing‐increasing class. For alcohol use, exposure to substance use in the household was associated with higher odds of belonging to the stable‐high trajectory relative to the stable‐low class, and no ACEs predicted membership in the stable‐high class compared to the increasing class. For drug use, only exposure to substance use in the household was associated with higher odds of belonging to the drug‐users trajectory relative to the nonusers class.

### Sensitivity analyses

In sensitivity analyses controlling for baseline PTSD symptoms, the results were largely consistent such that cumulative ACEs continued to predict deviant behavior and drug use trajectories; however, the association with the stable‐high alcohol use trajectory was attenuated and became nonsignificant, *p* = .071 (Supplemental Tables S3). The results from the simultaneous ACE models were unchanged (Supplementary Tables S4–S6).

## DISCUSSION

Results from this longitudinal study advance understanding of childhood adversity and externalizing symptom trajectories among U.S. National Guard personnel during early military service. Though many adults experience trauma, relatively few develop lasting psychopathology like PTSD (Kessler et al., 2017). Our findings show this pattern also applies to externalizing problems. We found that despite ACE exposure, most participants followed low‐risk developmental trajectories for deviant behavior, alcohol use, and drug use. However, higher cumulative ACE scores were associated with an increased risk of following more problematic trajectories across all domains, and specific childhood adversity types emerged as significant predictors of those trajectories when controlling for other ACEs.

The identification of distinct symptom trajectories highlights variability in adjustment during early military service. For deviant behavior, we found three trajectories: stable‐low (56.4%), decreasing‐increasing (21.4%), and increasing‐decreasing (22.2%). Notably, the two dynamic trajectories revealed different patterns of temporal change: Approximately one fifth of the sample showed initial improvement followed by later escalation (decreasing‐increasing), which may reflect initial compliance with military structure that erodes over time, whereas a similar proportion demonstrated the reverse pattern of initial escalation followed by improvement (increasing‐decreasing), potentially reflecting delayed adaptation to military norms or the positive effects of sustained exposure to structured institutional environments. The presence of these divergent patterns indicates substantial heterogeneity in how recruits respond during early military service (Salvatore & Taniguchi, 2021), with National Guard personnel potentially at an increased risk given their greater opportunity for deviant behavior compared to active duty service members (Gloyd & Leal, 2019). For alcohol use, over half of the participants exhibited concerning patterns, with 29.8% showing increasing and 24.6% exhibiting stable‐high use. For drug use, concerning patterns were less prevalent, with only 21.1% endorsing any drug use over time, likely reflecting the National Guard's strict zero‐tolerance drug policy. Alternatively, drug use may have been underreported given the zero‐tolerance policy and potential risks of reprisal despite assurances of anonymity.

Our study design, which began prior to BCT, provides unique insight into the distinction between preexisting symptoms and potentially military‐influenced trajectories. The initial assessment established baseline premilitary externalizing behaviors (Bonanno et al., 2015), allowing differentiation between individuals entering with elevated symptoms (stable‐high) versus those whose symptoms changed over the observation period. However, without a civilian control group, we cannot definitively attribute observed trajectory patterns to National Guard service itself versus normative developmental changes or other life experiences that occur during this period. The developmental timing offers a crucial perspective, as emerging adulthood— characterized by identity exploration and increased risky behaviors (Arnett, 2016; Schulenberg et al., 2018)—brings normative challenges that may influence externalizing behaviors independently of military service. For example, the increasing alcohol trajectory in nearly 30% of participants parallels broader developmental trends observed in civilian populations (Schulenberg et al., 2018), suggesting these patterns may reflect normative emerging adulthood experimentation rather than ACE‐ or military‐specific influences. Future research that incorporates matched civilian control groups is needed to disentangle the unique contributions of military service from normative developmental processes in shaping externalizing behavior trajectories.

We extend previous literature linking ACE exposure to internalizing psychopathology in military populations (Blosnich et al., 2021; Morgan et al., 2022; Slavin et al., 2024) by demonstrating parallel associations with externalizing symptom trajectories. Cumulative ACE scores showed distinct predictive patterns across domains. For deviant behavior, higher ACE scores predicted reduced odds of membership in the stable‐low trajectory, with no significant differences between the two more dynamic groups. This pattern suggests that although ACE exposure predicts elevated baseline deviant behavior, it does not differentially predict the specific direction or pattern of behavioral change during early military service. For alcohol use, ACE scores predicted membership in the stable‐high trajectory but not the increasing trajectory, and, for drug use, ACE scores predicted membership in the drug‐users trajectory relative to the nonusers class. These findings indicate that recruits with higher degrees of ACE exposure may enter military service with established elevated substance use rather than being at an increased risk for new substance use onset or use escalation during early service.

This study builds on prior research investigating specific ACEs and externalizing outcomes. Previous research identified sexual abuse and household incarceration as key predictors of substance problems in military populations (Clarke‐Walper et al., 2014; Moore et al., 2024), whereas civilian research has shown that both household dysfunction and abuse contribute to externalizing problems (Benjet et al., 2010). Consistent with this literature, physical abuse and domestic violence predicted increasing‐decreasing deviant behavior, whereas household substance use robustly predicted both stable‐high alcohol use and drug use endorsement even when controlling for all other ACEs. The association between early exposure to violence and an initial escalation of deviant behavior may reflect learned aggressive or antisocial patterns that emerge under stress but can be moderated over time within structured environments. Meanwhile, the pattern linking household substance use to persistent substance involvement suggests that exposure to familial substance use increases the general risk of substance problems rather than specific patterns of escalation, aligning with prior evidence linking familial substance use to early adult substance problems (Sebalo et al., 2023) and potentially reflecting a shared genetic liability for externalizing behavior (Wang et al., 2012).

Interestingly, our findings align more closely with civilian than military research. This similarity may be particularly relevant for National Guard “citizen soldiers” (Cohen et al., 2015), who spend considerably more time in civilian environments than active duty personnel. The absence of a significant association between ACEs and the increasing alcohol use trajectory suggests that other factors may play more prominent roles in behavioral escalation during this transition period, including unique reservist stressors and specific military experiences, as well as environmental factors, broader emerging adulthood influences, and individual differences in resilience. When compared to previous military research, our findings highlight how a trajectory‐based approach, which captures dynamic patterns rather than static associations, may reveal different associations and underscores the importance of considering developmental patterns when examining ACE effects, particularly during the transition into military service.

Nevertheless, we acknowledge several limitations. First, retrospective self‐report ACE assessment introduces potential recall bias (Hardt & Rutter, 2004), particularly for individuals with worse mental health (Martin‐Wagar et al., 2024). Second, the study used relatively coarse measures of adversity exposure; future research would benefit from dimensional assessments that consider timing, severity, and chronicity, as these factors are known to significantly influence outcomes related to childhood adversity (Hardi et al., 2025). Further, we substituted one item from the original 10‐item ACE questionnaire with a comparable item from another measure, which may limit direct comparability with studies that use the standard ACE scale. Third, the sample was drawn from a single state in the midwestern United States with a relatively young population, limiting generalizability to older military personnel and National Guard units from other geographic regions with different demographic compositions and regional characteristics. Fourth, the study experienced attrition, which is common in longitudinal research. However, attrition mainly occurred from pre‐ to post‐BCT and was low throughout the remaining follow‐up waves, which mitigates concern about sample bias.

We also acknowledge the limitations of our analytic approach. GMMs are sensitive to distributional characteristics and modeling decisions, and the emergence of similar three‐to‐four class solutions across studies may reflect methodological artifacts rather than true population heterogeneity (Sher et al., 2011). Replication of these trajectory patterns in independent samples is needed to establish their generalizability. Additionally, the moderate entropy (.619) for the deviant behavior model indicates classification uncertainty, which may affect the precision of effect size estimates and clinical utility of the identified classes. Our reliance on ACEs as the primary external validator is another limitation, as overlapping confidence intervals across pathological trajectory classes suggest modest predictor differentiation. Future research examining additional predictors (e.g., military stressors, social support) and distal outcomes is needed to more comprehensively validate these classes. Further, our separate analysis of the three externalizing domains precludes examining their shared variance. Although a single latent variable approach would have enhanced construct reliability and reduced multiple comparisons, it was precluded by significant measurement noninvariance across time points in our data. This noninvariance is itself a key finding, suggesting the behaviors do not coevolve uniformly. Thus, our method was a necessary analytical step that ultimately allowed us to discover the unique developmental pathway for each domain. Finally, this study did not explore potential mediators of the associations between ACEs and externalizing symptoms; thus, our findings are mechanism‐agnostic, and future research should examine potential pathways through which these associations may operate.

Despite these limitations, this study offers important theoretical and practical implications. Our findings demonstrate that both the quantity and type of ACE exposure predict divergent externalizing trajectories, with substantial portions of individuals who experienced childhood adversity experiencing stable‐low symptoms. The identification of distinct developmental pathways challenges deterministic models of adversity and emphasizes the need for nuanced approaches that account for both vulnerability and resilience. Practically, the results suggest that understanding recruits’ ACE histories may inform risk assessment and support strategies. Such assessment could be integrated into existing medical and psychological evaluations, allowing for risk stratification and the early identification of individuals who may benefit from additional support or monitoring. For individuals identified as higher‐risk, interventions could include enhanced mentorship programs, stress management training, or early referral to behavioral health services. However, given the modest effect sizes and substantial resilience observed among participants with ACE exposure, assessment approaches should focus on identifying modifiable risk factors and existing strengths rather than excluding individuals with adversity histories. Such approaches may enhance adaptive functioning during military service, potentially reducing attrition, improving readiness, and promoting long‐term well‐being.

## OPEN PRACTICES STATEMENT

This study was pre‐registered on January 22, 2025, and is available at https://osf.io/ybfex. The datasets used and/or analyzed during the current study are available from the principal investigator upon reasonable request at Polus002@umn.edu.

## AUTHOR NOTE

This work was supported by the National Center for Complementary & Integrative Health of the National Institutes of Health (NIH; UH3AT009651) and National Institute on Drug Abuse (NIDA; K01DA057359). This material was also supported with resources and the use of facilities at the Minneapolis Veterans Affairs (VA) Health Care System in Minneapolis, Minnesota. The content is solely the responsibility of the authors and does not necessarily represent the official views of the NIH, U.S. Department of the Army, U.S. Department of Defense, or the VA.

## AUTHOR CONTRIBUTIONS

**Ali F. Sloan**: conceptualization, methodology, formal analysis, writing ‐ original draft, visualization. **Tristan Bron**: conceptualization, writing ‐ review and editing. **Craig A. Marquardt**: conceptualization, writing ‐ review and editing. **Seth G. Disner**: conceptualization, writing ‐ review and editing. **Siamak Noorbaloochi**: conceptualization, writing ‐ review and editing, methodology, investigation. **Melissa A. Polusny**: conceptualization, investigation, funding acquisition, writing ‐ review and editing, project administration. **Jonathan D. Schaefer**: conceptualization, supervision, writing ‐ review and editing.

## Supporting information



SUPPORTING INFORMATION
