# Supplementary material for: Predicting externalizing symptom trajectories in U.S. National Guard recruits: The role of adverse childhood experiences
Source: J Trauma Stress. 2025 Dec 24;39(2):282–94. doi: 10.1002/jts.70037 (PMC13044390; doi:10.1002/jts.70037)
Supplement: Supplementary file 1 — SUPPORTING INFORMATION [file JTS-39-282-s001.pdf]

# Predicting externalizing symptom trajectories in U.S. National Guard recruits: The role of adverse childhood experiences

**Running head:** CHILDHOOD ADVERSITY AND EXTERNALIZING TRAJECTORIES

Ali F. Sloan,[1] Tristan Bron,[2] Craig A. Marquardt,[3,4] Seth G. Disner,[3,4] Siamak Noorbaloochi,[3,4,5] Melissa A. Polusny,[4] and Jonathan D. Schaefer[1]

[1]Department of Psychology, Vanderbilt University, Nashville, Tennessee, USA

[2]Department of Psychology, Liberty University, Lynchburg, Virginia, USA

[3]Minneapolis VA Health Care System, 1 Veterans Drive, Minneapolis, Minnesota, USA

[4]Department of Psychiatry and Behavioral Science, University of Minnesota, Minneapolis, Minnesota, USA

[5]Center for Care Delivery Outcomes Research, Minneapolis, Minnesota, USA

# Supplementary Method

## Detailed Growth Mixture Modeling Procedures

Multiple approaches exist for identifying latent trajectories in longitudinal data (for a survey see: Nguena Nguefack et al., 2020). We employed a systematic, multi-stage approach. First, we identified the optimal unconditional growth model for each externalizing domain, assuming a single class. Second, we used this optimal model structure as the basis for fitting Growth Mixture Models (GMM) with increasing numbers of classes.

To determine the most appropriate functional form of change over time, we compared eight single-class unconditional models per outcome. These models tested linear versus quadratic fixed effects for time and progressively more complex random effects structures. Each specification was then re-fit using a spline link, which models a flexible transformation between the underlying latent process and the observed outcome to better accommodate potential non-normality in the outcome distribution. The model with the lowest Bayesian Information Criterion (BIC) was selected as the base structure for GMM.

Using this best-fitting unconditional model structure, we then tested GMM solutions with one to four latent classes. This range is consistent with a recent systematic review of psychological symptom trajectories in military populations (Pavlacic et al., 2022), which frequently identifies between two and four classes. Common patterns include a resilience trajectory (stable-low symptoms) and symptomatic trajectories (e.g., stable-high, decreasing, increasing over time). For each class solution, models were tested with both homogeneous and heterogeneous within-class variance-covariance structures. Model selection was guided by multiple fit indices, including the BIC and Akaike Information Criterion (AIC). Clinical interpretability was also assessed based on minimum class size (≥5% of the sample), distinctiveness of the trajectories, and theoretical coherence with established developmental patterns of externalizing behaviors. In accordance with Nagin and Odgers' (2010) recommendations, which emphasize balancing statistical criteria with substantive theory and practical utility, we prioritized the model that offered optimal clinical relevance and interpretability.

We initially planned exploratory parallel process modeling of the three indicators simultaneously to examine co-evolution of externalizing behaviors, but the joint model failed to converge within an acceptable approximating error. Additionally, we tested measurement invariance of a latent externalizing factor across time points but found inadequate invariance, indicating the three domains did not load consistently onto a single factor over time. Given these psychometric limitations, we analyzed each externalizing domain separately to ensure model stability and interpretability, though we acknowledge this approach increases the number of statistical tests and may not capture the shared variance among externalizing behaviors.

To examine predictors of trajectory class membership, there are two competing methods: modeling the mixing probabilities within the mixture model (e.g., via multinomial cumulative logit model), or a two-step procedure of initial class assignment followed by uncertainty adjustment. The first approach has been implemented for parametric mixture models that are mostly mixtures of Gaussian or other well-behaved parametric models (e.g., Muthén, B., & Asparouhov, T. 2006), while the second approach is less model dependent. Here, we followed the second method.

After selecting the final GMM, to account for uncertainty in class assignment – particularly when posterior probabilities are similar across classes – we used estimated posterior probabilities of class membership to generate 10 imputed datasets. In each dataset, a participant was stochastically assigned to a single class by sampling based on their unique posterior probabilities. This creates 10 complete datasets with different, but probable, class assignments, rather than relying on a single, deterministic (most-likely) class assignment. We ran multinomial logistic regressions separately on each of the 10 imputed datasets, then pooled the results from these 10 regressions using Rubin's rules to produce a single set of estimates that properly accounts for class assignment uncertainty.

## Supplementary Results

### Detailed Model Selection

**Deviant Behavior.** The best-fitting unconditional (single-class) growth model included a quadratic fixed effect, random intercepts and linear slopes, and a splines link function (BIC = 4116). We then tested GMMs with increasing numbers of latent classes and found that model fit improved. Of the multi-class solutions, a three-class model provided the optimal fit (BIC = 3916) and demonstrated adequate classification certainty (entropy = 0.619), indicating moderate separation between classes. This selected 3-class GMM provided a substantially better fit than the best-fitting unconditional growth model (ΔBIC = 200), justifying the use of a mixture modeling approach.

**Alcohol Use.** The best-fitting unconditional growth model included a quadratic fixed effect, random intercepts with linear and quadratic slopes, and a splines link function (BIC = 3822). When multi-class solutions were tested, a three-class model with varying within-group variability across classes yielded the lowest BIC (BIC = 3403). Moreover, the model demonstrated reasonable classification certainty (entropy = 0.758), suggesting adequate separation between the identified classes. The final 3-class GMM (BIC = 3403) provided a substantially better fit than the optimal unconditional growth model (ΔBIC = 419), strongly supporting the mixture modeling approach.

**Drug Use.** The best-fitting unconditional growth model included a quadratic fixed effect, random intercepts with linear and quadratic slopes, and a splines link function (BIC = 513). Testing multi-class solutions, we found that a two-class model with varying within-group variability across classes yielded the best statistical fit, as indicated by the lowest BIC (BIC = -1726). This model also demonstrated excellent classification certainty (entropy = 0.974), indicating clear separation between classes. Given the substantial statistical superiority of this solution and its meaningful distinction between consistent non-users and those with any drug use engagement—a clinically relevant differentiation given the military's zero-tolerance drug policy—we advanced this model over more complex three- or four-class solutions. The selected 2-class GMM provided a substantially better fit than the best unconditional growth model (ΔBIC = 2,239), supporting the mixture modeling approach.

**Table S1.** ***Fit statistics for the unconditional models for externalizing symptoms across assessment waves***

| | Trajectory Type | Random Effects | Link Function | Log Likelihood | BIC |
|---|---|---|---|---|---|
| Deviant Behavior | | | | | |
| | Linear | Intercept | Linear | -4072.541 | 8171.327 |
| | Linear | Intercept, Slope | Linear | -4029.197 | 8097.761 |
| | Quadratic | Intercept, Slope | Linear | -4028.989 | 8103.906 |
| | Quadratic | Intercept, Slope, Curvature | Linear | -4000.972 | 8067.553 |
| | Linear | Intercept | Splines | -2033.285 | 4125.619 |
| | Linear | Intercept, Slope | Splines | -2024.094 | 4120.359 |
| | **Quadratic** | **Intercept, Slope** | **Splines** | **-2018.482** | **4115.697** |
| | Quadratic | Intercept, Slope, Curvature | Splines | -2014.108 | 4126.631 |
| Alcohol Use | | | | | |
| | Linear | Intercept | Linear | -1000000000 | 2000000026.216 |
| | Linear | Intercept, Slope | Linear | -3780.382 | 7600.087 |
| | Quadratic | Intercept, Slope | Linear | -1000000000 | 2000000045.878 |
| | Quadratic | Intercept, Slope, Curvature | Linear | -1000000000 | 2000000065.539 |
| | Linear | Intercept | Splines | -2006.330 | 4071.645 |
| | Linear | Intercept, Slope | Splines | -1953.017 | 3978.127 |
| | Quadratic | Intercept, Slope | Splines | -1897.029 | 3872.705 |
| | **Quadratic** | **Intercept, Slope, Curvature** | **Splines** | **-1862.072** | **3822.454** |
| Drug Use | | | | | |
| | Linear | Intercept | Linear | -4174.897 | 8376.033 |
| | Linear | Intercept, Slope | Linear | -4154.992 | 8349.342 |
| | Quadratic | Intercept, Slope | Linear | -4154.779 | 8355.475 |
| | Quadratic | Intercept, Slope, Curvature | Linear | -4149.153 | 8363.903 |
| | Linear | Intercept | Splines | -240.100 | 539.236 |
| | Linear | Intercept, Slope | Splines | -228.331 | 528.818 |
| | Quadratic | Intercept, Slope | Splines | -223.869 | 526.453 |
| | **Quadratic** | **Intercept, Slope, Curvature** | **Splines** | **-207.549** | **513.492** |

Note. BIC = Bayesian Information Criterion. AIC = Akaike's Information Criterion. Bold indicates the selected model.

**Table S2.** ***Fit statistics for the growth mixture modelling for externalizing symptoms across assessment waves***

| | No. Classes | Variance Structure | Log likelihood | BIC | AIC | Entropy | Min. Class Size |
|---|---|---|---|---|---|---|---|
| Deviant Behavior | | | | | | | |
| | 1 | - | -2018.482 | 4115.697 | 4060.965 | - | - |
| | 2 | Homogeneous | -1949.791 | 4004.559 | 3931.582 | 0.767 | 20.37% |
| | 2 | Heterogeneous | -1925.698 | 3962.934 | 3885.397 | 0.600 | 40.45% |
| | 3 | Homogeneous | -1927.779 | 3986.778 | 3895.558 | 0.758 | 5.94% |
| | **3** | **Heterogeneous** | **-1885.616** | **3915.574** | **3815.231** | **0.619** | **21.36%** |
| | 4 | Homogeneous | -1927.769 | 4013.003 | 3903.539 | 0.503 | 0.00% |
| | 4 | Heterogeneous | -1885.635 | 3948.417 | 3825.270 | 0.443 | 0.00% |
| Alcohol Use | | | | | | | |
| | 1 | - | -1862.072 | 3822.454 | 3754.145 | | |
| | 2 | Homogeneous | -1791.802 | 3708.128 | 3621.603 | 0.751 | 42.74% |
| | 2 | Heterogeneous | -1650.414 | 3431.907 | 3340.828 | 0.793 | 47.15% |
| | 3 | Homogeneous | -1791.801 | 3734.343 | 3629.603 | 0.602 | 0.00% |
| | **3** | **Heterogeneous** | **-1619.702** | **3403.252** | **3289.404** | **0.758** | **24.64%** |
| | 4 | Homogeneous | -1712.368 | 3601.693 | 3478.736 | 0.564 | 0.00% |
| | 4 | Heterogeneous | -1635.803 | 3468.224 | 3331.606 | 0.611 | 0.00% |
| Drug Use | | | | | | | |
| | 1 | - | -207.549 | 513.492 | 445.097 | N/A | |
| | 2 | Homogeneous | 83.736 | -42.838 | -129.471 | 0.954 | 8.78% |
| | **2** | **Heterogeneous** | **928.672** | **-1726.151** | **-1817.344** | **0.974** | **21.10%** |
| | 3 | Homogeneous | 223.495 | -296.119 | -400.990 | 0.964 | 4.53% |
| | 3 | Heterogeneous | 928.670 | -1693.350 | -1807.340 | 0.952 | 0.00% |
| | 4 | Homogeneous | 423.210 | -669.311 | -792.420 | 0.791 | 0.00% |
| | 4 | Heterogeneous | 928.646 | -1660.504 | -1797.293 | 0.588 | 0.00% |

Note. No. Classes = Number of classes. BIC = Bayesian Information Criterion. AIC = Akaike's Information Criterion. Min. Class Size = Minimum class size. Bold indicates the selected model.

**Table S3.** ***Cumulative ACE Score Predicting Trajectory Class Membership Controlling for PTSD***

| | Class | OR | 95% CI | *p* value |
|---|---|---|---|---|
| Deviant Behavior | | | | |
| | Decreasing-Increasing | **1.22** | **(1.07-1.39)** | **.004** |
| | Increasing-Decreasing | **1.22** | **(1.12-1.34)** | **<.001** |
| Alcohol Use | | | | |
| | Increasing | 1.02 | (0.93-1.13) | .652 |
| | Stable-High | 1.10 | (0.99-1.21) | .071 |
| Drug Use | | | | |
| | Drug Users | **1.19** | **(1.10-1.28)** | **<.001** |

Note. Results from multinomial logistic regression models with cumulative ACE score predicting class membership, pooled across 10 multiply imputed datasets using Rubin's rules. All models are adjusted for age, sex, race and baseline PTSD symptoms. Reference category is the stable-low trajectory class for all analyses. Values greater than 1 indicate a higher likelihood of trajectory group membership associated with cumulative ACEs. OR = odds ratio; CI = confidence interval. Significant associations ($p < .05$) are indicated in bold.

**Table S4.** ***Individual ACEs Predicting Deviant Behavior Trajectory Class Membership Controlling for PTSD***

| | | Individual | | | Simultaneous | | |
|---|---|---|---|---|---|---|---|
| | ACE | OR | 95% CI | *p* value | OR | 95% CI | *p* value |
| Decreasing-Increasing | | | | | | | |
| | DV | **2.32** | **(1.25-4.34)** | **.009** | 1.69 | (0.88-3.24) | .112 |
| | EA | **2.13** | **(1.24-3.66)** | **.007** | 1.24 | (0.64-2.41) | .528 |
| | EN | **1.68** | **(1.01-2.79)** | **.045** | 1.04 | (0.59-1.85) | .888 |
| | IN | 1.77 | (0.76-4.11) | .181 | 1.06 | (0.41-2.72) | .900 |
| | MI | 1.70 | (0.85-3.38) | .128 | 1.08 | (0.5-2.34) | .832 |
| | PA | **2.36** | **(1.31-4.27)** | **.005** | 1.50 | (0.71-3.14) | .284 |
| | PL | 1.54 | (0.81-2.92) | .179 | 1.10 | (0.56-2.14) | .782 |
| | PN | 1.98 | (0.85-4.59) | .110 | 1.06 | (0.42-2.69) | .900 |
| | SA | 1.77 | (0.82-3.82) | .147 | 1.14 | (0.49-2.63) | .756 |
| | SU | **2.07** | **(1.08-3.97)** | **.029** | 1.39 | (0.72-2.66) | .323 |
| Increasing-Decreasing | | | | | | | |
| | DV | **2.9** | **(1.8-4.67)** | **<.001** | **2.38** | **(1.32-4.27)** | **.004** |
| | EA | **1.92** | **(1.21-3.05)** | **.006** | 0.80 | (0.4-1.61) | .527 |
| | EN | **1.65** | **(1.05-2.59)** | **.030** | 1.04 | (0.57-1.89) | .909 |
| | IN | 1.88 | (0.93-3.81) | .078 | 1.16 | (0.49-2.74) | .734 |
| | MI | 1.50 | (0.92-2.43) | .101 | 0.99 | (0.56-1.77) | .982 |
| | PA | **2.99** | **(1.73-5.19)** | **<.001** | **2.42** | **(1.2-4.9)** | **.014** |
| | PL | 1.22 | (0.76-1.95) | .408 | 0.80 | (0.45-1.43) | .442 |
| | PN | 1.85 | (0.81-4.24) | .144 | 0.92 | (0.36-2.34) | .865 |
| | SA | 1.60 | (0.73-3.49) | .239 | 1.07 | (0.45-2.55) | .884 |
| | SU | **2.18** | **(1.33-3.59)** | **.002** | 1.60 | (0.86-2.95) | .134 |

Note. Results from multinomial logistic regression models examining associations between individual ACE exposures and deviant behavior trajectory class membership, pooled across 10 multiply imputed datasets using Rubin's rules. Individual models show results when each ACE is entered separately; simultaneous models show results when all ACEs are entered together. All models are adjusted for age, sex, race, and baseline PTSD symptoms. Reference category is the stable-low trajectory class. Values greater than 1 indicate a higher likelihood of trajectory group membership associated with the corresponding ACE. ACE types: DV = domestic violence; EA = emotional abuse; EN = emotional neglect; IN = household incarceration; MI = household mental illness; PA = physical abuse; PL = parental loss; PN = physical neglect; SA = sexual abuse; SU = household substance use. OR = odds ratio; CI = confidence interval. Significant associations ($p < .05$) are indicated in bold.

**Table S5.** ***Individual ACEs Predicting Alcohol Use Trajectory Class Membership Controlling for PTSD***

| | | Individual | | | Simultaneous | | |
|---|---|---|---|---|---|---|---|
| | ACE | OR | 95% CI | *p* value | OR | 95% CI | *p* value |
| Increasing | | | | | | | |
| | DV | 1.09 | (0.67-1.75) | .736 | 1.01 | (0.59-1.71) | .980 |
| | EA | 1.10 | (0.7-1.72) | .691 | 0.93 | (0.49-1.75) | .814 |
| | EN | 1.25 | (0.8-1.95) | .323 | 1.24 | (0.72-2.12) | .438 |
| | IN | 0.89 | (0.4-1.94) | .759 | 0.83 | (0.35-1.96) | .670 |
| | MI | 1.00 | (0.57-1.75) | .997 | 0.99 | (0.51-1.9) | .972 |
| | PA | 1.21 | (0.73-1.98) | .461 | 1.26 | (0.68-2.34) | .458 |
| | PL | 1.24 | (0.83-1.85) | .292 | 1.24 | (0.8-1.93) | .333 |
| | PN | 0.82 | (0.4-1.67) | .583 | 0.76 | (0.34-1.67) | .487 |
| | SA | 0.56 | (0.2-1.58) | .265 | 0.53 | (0.19-1.51) | .229 |
| | SU | 1.08 | (0.62-1.87) | .794 | 1.08 | (0.56-2.08) | .825 |
| Stable-High | | | | | | | |
| | DV | 1.5 | (0.92-2.45) | .100 | 1.32 | (0.75-2.33) | .339 |
| | EA | 1.35 | (0.79-2.3) | .271 | 0.85 | (0.43-1.7) | .654 |
| | EN | 1.38 | (0.84-2.26) | .202 | 1.16 | (0.65-2.08) | .620 |
| | IN | 1.33 | (0.68-2.61) | .402 | 0.96 | (0.46-2.02) | .911 |
| | MI | 1.27 | (0.77-2.08) | .343 | 0.99 | (0.55-1.79) | .971 |
| | PA | 1.53 | (0.86-2.71) | .146 | 1.41 | (0.73-2.75) | .308 |
| | PL | 1.26 | (0.82-1.94) | .296 | 1.03 | (0.64-1.66) | .908 |
| | PN | 0.74 | (0.36-1.55) | .403 | 0.46 | (0.2-1.05) | .064 |
| | SA | 0.89 | (0.4-1.97) | .776 | 0.76 | (0.34-1.72) | .511 |
| | SU | **1.97** | **(1.13-3.44)** | **.017** | **2.03** | **(1.03-4.02)** | **.042** |

Note. Results from multinomial logistic regression models examining associations between individual ACE exposures and alcohol use trajectory class membership, pooled across 10 multiply imputed datasets using Rubin's rules. Individual models show results when each ACE is entered separately; simultaneous models show results when all ACEs are entered together. All models are adjusted for age, sex, race, and baseline PTSD symptoms. Reference category is the stable-low trajectory class. Values greater than 1 indicate a higher likelihood of trajectory group membership associated with the corresponding ACE. ACE types: DV = domestic violence; EA = emotional abuse; EN = emotional neglect; IN = household incarceration; MI = household mental illness; PA = physical abuse; PL = parental loss; PN = physical neglect; SA = sexual abuse; SU = household substance use. OR = odds ratio; CI = confidence interval. Significant associations ($p < .05$) are indicated in bold.

**Table S6.** ***Individual ACEs Predicting Drug Users Trajectory Class Membership Controlling for PTSD***

| | Individual | | | Simultaneous | | |
|---|---|---|---|---|---|---|
| ACE | OR | 95% CI | *p* value | OR | 95% CI | *p* value |
| DV | **1.87** | **(1.24-2.81)** | **.003** | 1.46 | (0.91-2.34) | .120 |
| EA | **1.80** | **(1.21-2.69)** | **.004** | 0.82 | (0.46-1.48) | .515 |
| EN | **1.99** | **(1.34-2.97)** | **<.001** | 1.47 | (0.91-2.37) | .119 |
| IN | 1.36 | (0.78-2.37) | .275 | 0.83 | (0.45-1.54) | .562 |
| MI | **1.56** | **(1.03-2.36)** | **.035** | 1.04 | (0.63-1.71) | .870 |
| PA | **2.28** | **(1.47-3.54)** | **<.001** | 1.74 | (0.98-3.09) | .060 |
| PL | 1.12 | (0.77-1.65) | .554 | 0.78 | (0.5-1.2) | .257 |
| PN | 1.68 | (0.93-3.03) | .088 | 0.92 | (0.47-1.78) | .797 |
| SA | **1.91** | **(1.02-3.58)** | **.045** | 1.48 | (0.76-2.89) | .254 |
| SU | **2.27** | **(1.5-3.43)** | **<.001** | **1.86** | **(1.12-3.1)** | **.017** |

Note. Results from multinomial logistic regression models examining associations between individual ACE exposures and drug-users trajectory class membership, pooled across 10 multiply imputed datasets using Rubin's rules. Individual models show results when each ACE is entered separately; simultaneous models show results when all ACEs are entered together. All models are adjusted for age, sex, and race. Reference category is the non-users trajectory class. Values greater than 1 indicate a higher likelihood of trajectory group membership associated with the corresponding ACE. ACE types: DV = domestic violence; EA = emotional abuse; EN = emotional neglect; IN = household incarceration; MI = household mental illness; PA = physical abuse; PL = parental loss; PN = physical neglect; SA = sexual abuse; SU = household substance use. OR = odds ratio; CI = confidence interval. Significant associations ($p < .05$) are indicated in bold.

**Figure S1. CONSORT Diagram**

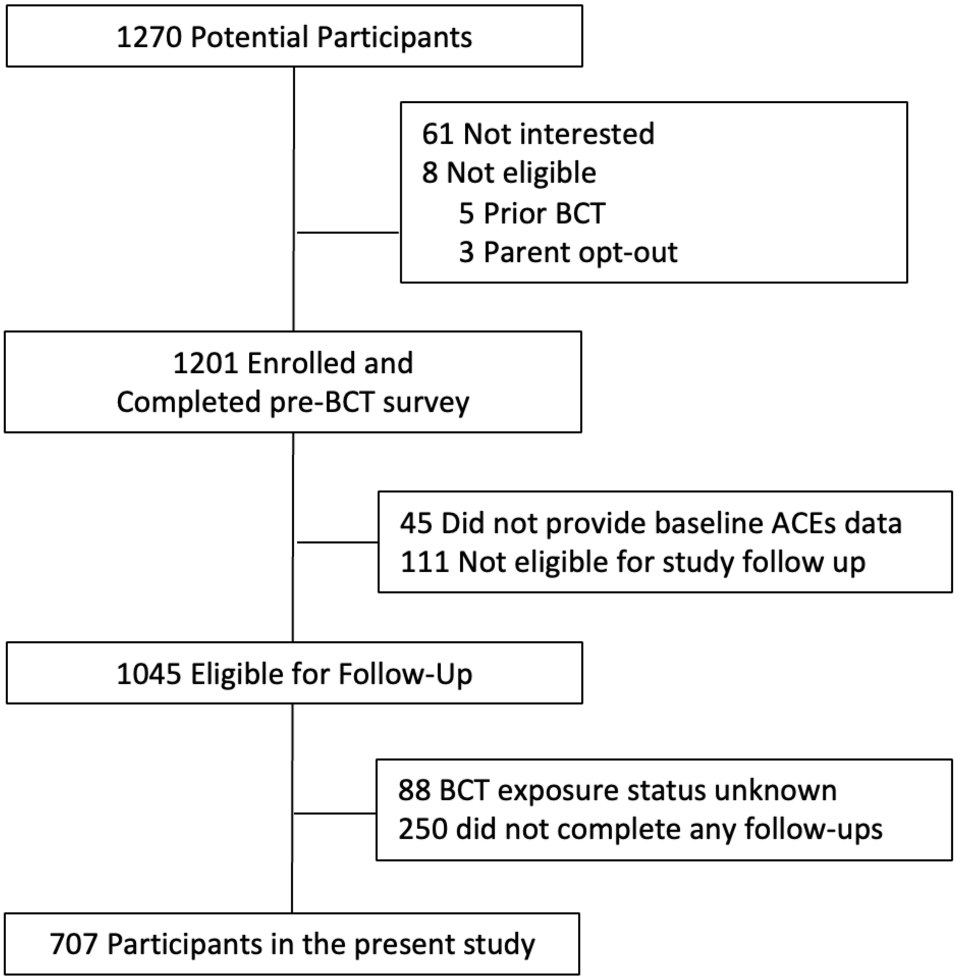

**Figure S2. Prevalence of Adverse Childhood Experiences**

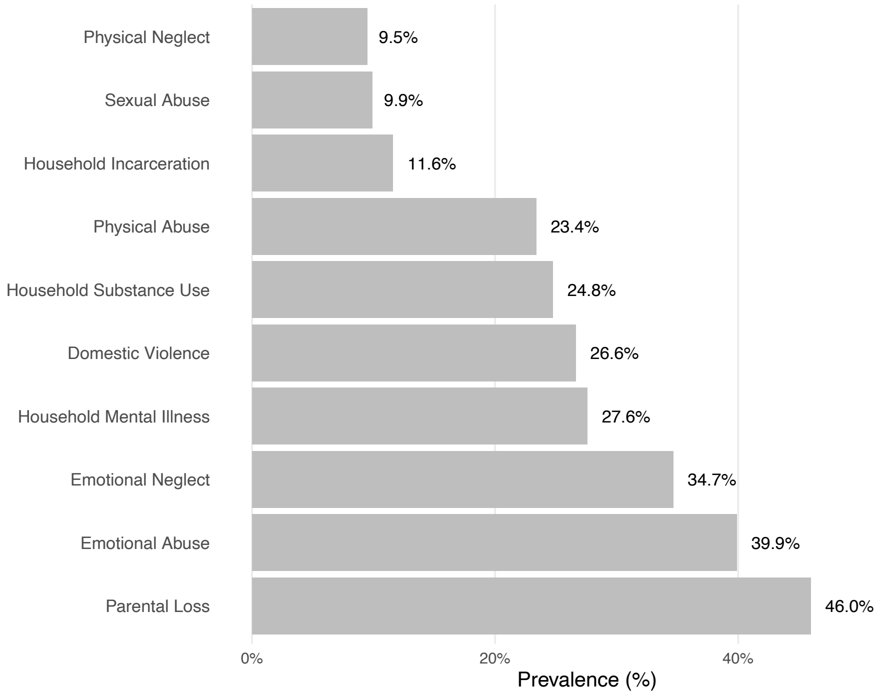


Note. Percentage of the analytic sample exposed to each specific ACE.
